# Chromatin and transcriptional dynamics underlying the immune-modulatory effects of vitamin D_3_ in vivo

**DOI:** 10.1038/s41598-025-32831-z

**Published:** 2025-12-18

**Authors:** Maciej Rybiński, Ranjini Ghosh Dastidar, Natalia Zawrotna, Emilia Gospodarska, Carsten Carlberg

**Affiliations:** 1 Institute of Animal Reproduction and Food Research, Polish Academy of Sciences, ul. Trylińskiego 18, 10-683 Olsztyn, Poland; 2 Institute of Biomedicine, University of Eastern Finland, Kuopio, Finland; 3 Present Address: Department of Medical Chemistry, Medical University of Gdansk, Gdansk, Poland

**Keywords:** Vitamin D, Chromatin accessibility, Transcriptome, Responsive genes, Immune system, Computational biology and bioinformatics, Genetics, Immunology, Molecular biology

## Abstract

Vitamin D_3_ is an essential micronutrient that supports innate immunity and modulates adaptive responses, with in vitro studies implicating epigenetic mechanisms. Yet, in vivo evidence for such regulation remains scarce. Here, we assessed genome-wide epigenomic and transcriptomic responses to vitamin D_3_ in a high responder from the VitDHiD repeated-measures, non-randomized interventional N-of-1 study, in which the participant received monthly oral boluses of 80,000 IU vitamin D_3_ over three months. Peripheral blood mononuclear cells (PBMCs) were collected at baseline (day 0) and at 24 and 48 h post-supplementation (days 1 and 2) for ATAC-seq and RNA-seq profiling. ATAC-seq identified > 3,500 regions with increased chromatin accessibility, predominantly at promoters, indicating a strong epigenomic activation. Motif enrichment revealed immune-regulatory transcription factors, but not vitamin D receptor (VDR) motifs, suggesting indirect or cooperative modes of regulation. RNA-seq detected 380 vitamin D–responsive genes with time-dependent expression changes, including *DUSP6* and *FOS*, enriched in pathways related to innate immunity and interferon signaling. Integrative analysis linked 306 differentially expressed genes to vitamin D–sensitive chromatin regions. Many of these overlap with VDR binding sites or promoters of neighboring non-target genes, suggesting potential long-range regulation. Shared enhancers and promoters among neighboring genes indicated redundancy in regulatory architecture. Analysis of PBMCs from 13 participants in the VitDPAS non-randomized interventional cohort study, each receiving a single vitamin D_3_ bolus, confirmed the main findings from the N-of-1 study but also revealed pronounced inter-individual variability in epigenomic and transcriptomic responses. Together, these results provide in vivo evidence that vitamin D signaling directly and indirectly modulates chromatin accessibility and gene expression, emphasizing its role as an epigenetic regulator of immune function and supporting its potential in personalized nutrition and immune health strategies.

**Supplementary Information:**

The online version contains supplementary material available at 10.1038/s41598-025-32831-z.

## Introduction

Vitamin D_3_ is an essential micronutrient produced in the skin via UV-B radiation^1^ or obtained through diet and supplementation^2^. Its most recognized role is in calcium homeostasis, crucial for bone mineralization^3^, yet evolutionary evidence points to earlier functions in cellular energy balance and detoxification^4–6^. Beyond skeletal health, vitamin D_3_ exerts potent immunomodulatory effects^7–12^: it boosts innate immune defense against pathogens such as *Mycobacterium tuberculosis*^13^ and SARS-CoV-2^14^ while attenuating excessive adaptive responses^15^. This dual action is critical for preventing autoimmune diseases like multiple sclerosis^16,17^ and reducing hyperinflammation in severe viral infections^18^. These effects are mediated by its active form, 1α,25-dihydroxyvitamin D_3_ (1,25(OH)_2_D_3_), which binds with high affinity (K_D_ ≈ 0.1 nM) to the vitamin D receptor (VDR)^19–21^, a nuclear receptor^22^ that modulates gene expression through direct genomic binding^23^. VDR is expressed in most tissues except the brain (GTEx data^24^) and regulates hundreds of genes in a tissue- and individual-specific manner^25,26^. This variability underlies the concept of the vitamin D response index, which classifies individuals as high, mid, or low responders to supplementation, an important factor for optimizing clinical outcomes^27^.

At the molecular level, chromatin immunoprecipitation sequencing (ChIP-seq) has mapped the VDR cistrome in various in vitro models, including THP-1 monocytic leukemia cells^28^. More than 10,000 potential VDR binding sites have been identified^29^, yet only a few hundred are consistently occupied across contexts^30^. DNA binding of VDR is stabilized by the retinoid X receptor (RXR)^31^. VDR–RXR heterodimers preferentially recognize DR3-type motifs, which are direct repeats separated by three nucleotides containing the AGTTCA hexamer^32^, but alternative DNA-binding configurations also occur. VDR may bind cooperatively with other nuclear proteins^33–35^ or indirectly via “piggybacking” on DNA-bound transcription factors^36^. Notably, VDR binding sites often co-localize with motifs for the myeloid cell pioneer factor PU.1, which is enriched under many VDR peaks^29^; about two-thirds of VDR binding regions also display PU.1 occupancy^37^. This is biologically plausible given the roles of PU.1, VDR, and the pioneer factor CEBPα^38^ in hematopoiesis, directing myeloid progenitors toward monocyte and granulocyte lineages^39^. Additional transcription factors such as GABPα^40^ and ETS1^41^ also co-localize with VDR, suggesting broader cooperative networks. Furthermore, cell type–specific pioneer factors, including CEBPα and RUNX2 in osteoblasts^42^ and BACH2 in T cells^43^, further emphasize the context-dependent nature of vitamin D signaling.

Chromatin structure varies extensively across the genome^44^ and critically influences transcription factor accessibility^45^. Genome-wide accessibility can be profiled by DNase-seq^46^, FAIRE-seq^47^, or ATAC-seq^48^. Most of the genome exists as compact heterochromatin, which is transcriptionally silent and structurally protective, whereas accessible euchromatic regions permit transcription factor binding in a cell- and tissue-specific manner. This organization is directly relevant to vitamin D signaling, as VDR binds only at open regulatory elements, restricting its activity to genes whose promoters lie in accessible regions. A chromatin-based model of vitamin D signaling^49^ proposes that VDR-bound enhancers act within the same topologically associating domain (TAD) as their target genes^29,50,51^, with TADs, which are spanning 100 kb to 2 Mb, defining the boundaries for enhancer–promoter communication^52^.

PBMCs, easily obtained and containing both innate and adaptive immune cell types, provide a suitable model for studying vitamin D effects in vivo^53^. Within PBMCs, monocytes are the most responsive to vitamin D signaling^54^. Here, we profiled epigenomic and transcriptomic changes in PBMCs from a healthy donor at 24 and 48 h after an oral vitamin D_3_ bolus, using three biological replicates. We then compared these results with epigenomic responses in a cohort of 13 participants from the VitDPAS trial, each of whom received a single vitamin D_3_ bolus.

## Materials and methods

### Participants and ethical approval

Peripheral blood samples were obtained from a single healthy male participant (age 59) enrolled in the VitDHiD study (ClinicalTrials.gov NCT03537027, registered on April 26, 2018) in Kuopio, Finland, who had previously been classified as a high responder to vitamin D_3_ supplementation^55^. VitDHiD has a repeated-measures, non-randomized interventional N-of-1 design involving monthly bolus supplementation with vitamin D_3_. In the VitDPAS study (ClinicalTrials.gov NCT06104111, registered on October 19, 2023), having a non-randomized interventional cohort design, healthy individuals from Olsztyn, Poland, received a body weight-adjusted bolus dose of vitamin D_3_ (1,000 IU/kg)^56^. We selected a cohort of 13 representative individals (5 females, 8 males, four high responders, five mid responders, and four low responders).

The study protocol of the VitDHiD study was approved by the Ethics Committee of the Northern Savo Hospital District (approval no. 515/2018), while the study protocol of the VitDPAS study was approved by the Ethics Committee of the Olsztyn Chamber of Physicians (approval no. 31/2023/VIII). All 14 participants provided written informed consent to participate in the study. All procedures were conducted in accordance with the Declaration of Helsinki.

For the N-of-1 approach the vitamin D status was measured using UPLC (1290 Infinity II LC System, Agilent) coupled with MS detection (API 4000 LC–MS/MS System, SCIEX)^57^, while for the cohort approch serum 25(OH)D_3_ levels were assayed using the electrochemiluminescence binding assay on Cobas Pure Immunoassay Analyzer (Roche) in the analytical laboratory of the Municipal Polyclinical Hospital in Olsztyn, Poland^56^.

### PBMC isolation

Blood (8 ml) was collected immediately before (day 0 (d0)), and at 24 h (d1) and 48 h (d2) following the oral vitamin D_3_ bolus. For the VitDPAS cohort blood was collected only on d0 and d1. Within one hour of blood collection, PBMCs were isolated using Vacutainer CPT Cell Preparation Tubes with sodium citrate (Becton Dickinson), following the manufacturer’s instructions. Isolated cells were washed with phosphate-buffered saline, aliquoted at a concentration of 4 × 10^6^ cells/ml, and stored at –80 °C for subsequent chromatin accessibility and transcriptomic analyses. For the N-of-1 approach, this protocol was repeated over three consecutive months using the same individual as the donor.

### ATAC-seq and RNA-seq library preparation and sequencing

For ATAC-seq, nuclei were isolated from 100,000 PBMCs by incubation in 50 µl lysis buffer (10 mM Tris–HCl, pH 8.0; 10 mM NaCl; 3 mM MgCl₂; 0.1% Tween-20; 0.1% IGEPAL CA-630; 0.01% digitonin). After 3 min on ice, 1 ml of wash buffer (10 mM Tris–HCl, pH 8.0; 10 mM NaCl; 3 mM MgCl₂; 0.1% Tween-20) was added. Nuclei were pelleted and subjected to transposition using a Tn5 transposase reaction mix (Illumina), supplemented with 0.01% digitonin and 0.1% Tween-20. The transposition reaction was carried out at 37 °C for 30 min in a thermomixer. Transposed DNA was purified using the DNA Clean & Concentrator-5 Kit (Zymo Research), followed by PCR amplification, purification, and size selection with SPRISelect beads (Beckman Coulter). Library quality was assessed using an Agilent TapeStation. Indexed libraries were quantified, pooled, and prepared for sequencing. For RNA-seq, total RNA was extracted from PBMCs using the High Pure RNA Isolation Kit (Roche), following the manufacturer’s instructions. RNA quality was evaluated on an Agilent TapeStation. Ribosomal RNA was depleted using kits from New England Biolabs, and libraries were prepared according to the manufacturer’s protocols. ATAC-seq and RNA-seq libraries were sequenced with 75 bp paired-end reads on a NextSeq 2000 platform (Illumina) at the EMBL GeneCore Facility (Heidelberg, Germany), following standard protocols.

### Epigenome analysis

Quality control of raw sequencing data was performed using FastQC (v0.12.0; www.bioinformatics.babraham.ac.uk/projects/fastqc). Adapter sequences were removed using TrimGalore (v0.6.10). Paired-end reads were aligned to the human reference genome GRCh38.p14 using the STAR aligner (v2.7.2a) with default parameters unless stated otherwise. Read mapping statistics are summarized in Table S1. Reads aligning to ENCODE blacklisted regions or with low mapping quality scores (MAPQ < 2) were filtered using the samtools view function with the -q 2 parameter. PCR duplicates were removed using samtools markdup (v1.9) to reduce amplification bias. Peak calling was performed with MACS3 (v3.0.1) using the callpeak function and the following parameters: -f BAMPE –verbose 3 –shift 0 -g hs -q 0.01 -B. Peak overlap analysis was conducted with bedtools (v2.30.0) and bedops (v2.4.41). Principal component analysis (PCA) was used to assess the global structure of the dataset, and MA (Mean Average) plots were generated using ggplot2 (v3.5.1; https://ggplot2.tidyverse.org). Peak heatmaps were produced with the plotHeatmap function from the deepTools package^58^ (v3.5.6), using coordinates of significant peaks identified in the consensus ATAC-seq dataset. Regions of accessible chromatin were visualized using the Integrative Genomics Viewer (IGV)^59,60^.

### Differential chromatin accessibility analysis

Differential chromatin accessibility analysis was conducted in R (v4.4.0) on macOS 12.6 using the DiffBind package^61^ (v3.8.4). A consensus peak set was generated using the dba.count function with the summits parameter set to 75, followed by merging of overlapping summits. This process yielded a consensus set of 33,836 peak summits for the N-of-1 approach (Table S2) and 23,945 peak summits for the cohort approach (Table S3). Peak annotation was performed using the ChIPseeker package (v1.40.0), in combination with the TxDb.Hsapiens.UCSC.hg38.knownGene annotation database (v3.16.0) and the org.Hs.eg.db package (v3.16.0). Differential accessibility was evaluated through pairwise comparisons between treatment time points (d1 versus d0 and d2 versus d0), using DESeq2-based functions integrated within DiffBind. In the N-of-1 approach, peaks were considered differentially accessible if they met the following criteria: a false discovery rate (FDR) < 0.1 in at least one comparison and an average signal intensity > 250, as determined by the dba.report function. In the cohort approach, genomic regions were considered differentially regulated if the p-value was below 0.05. Motif enrichment analysis for differentially accessible regions was conducted using HOMER (Hypergeometric Optimization of Motif EnRichment) software^62^ (version 5.1).

### Transcriptome analysis

Quality control of RNA-seq data was performed using FastQC. Single-end, reverse-stranded cDNA reads were aligned to the GRCh38.p14 human reference genome (GENCODE release 43) using the STAR aligner (v2.7.2a). Read quantification was carried out with FeatureCounts (v2.0.1). Gene annotation, including Ensembl gene identifiers, gene symbols, descriptions, genomic coordinates, biotypes, and Entrez Gene IDs, was retrieved from the Ensembl database (release 100) using the R packages biomaRt (v2.54.1) and org.Hs.eg.db (v3.16.0). Genes lacking HGNC symbols or encoded by the mitochondrial genome were excluded. For downstream analyses, only protein-coding genes were retained.

### Differential gene expression analysis

Differential gene expression analysis was conducted in R (v4.4.0) using the DESeq2 package^61^ (v1.44.0). A dataset comprising raw counts for 11,743 expressed genes (CPM > 10), along with corresponding metadata, was used to construct the DESeq2 object (Table S4). Differential expression between treatment groups (d1 versus d0 and d2 versus d0) was assessed using the Wald test. Genes were initially considered differentially expressed based on an unadjusted p-value < 0.05. To enhance stringency, a second filtering step was applied using a FDR threshold of 0.05, retaining only those genes meeting both significance criteria.

## Results

### Epigenome-wide response to vitamin D_3_ bolus supplementation

**Fig. 1:**
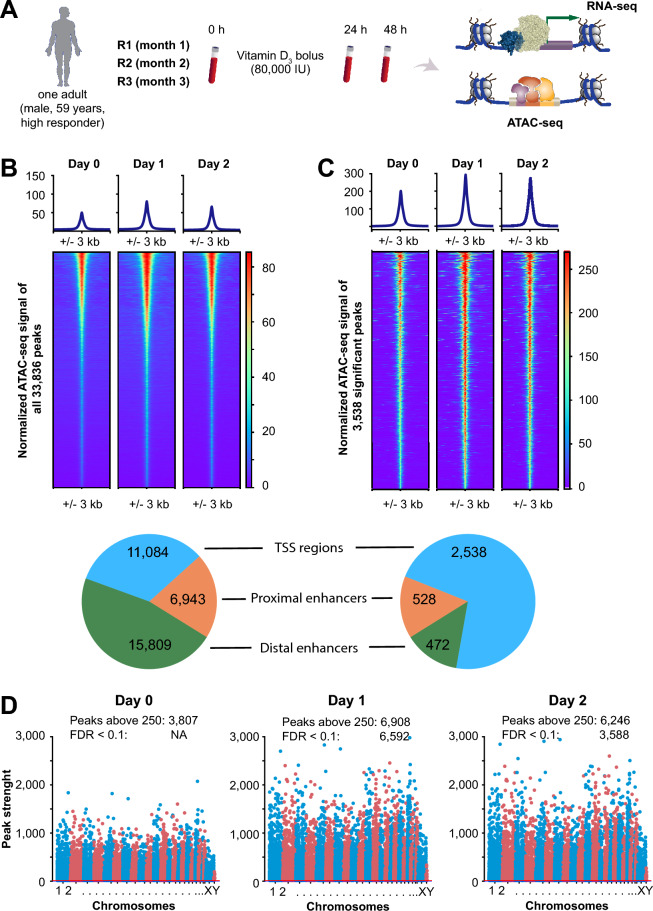
Assessment of Chromatin Accessibility In Response To Vitamin D_3_. Schematic representation of the experimental workflow (**A**). The in vivo study was conducted in three biological replicates (R1–R3). Heatmaps showing ATAC-seq signal intensities for all consensus peaks across days 0, 1, and 2 post-supplementation, illustrating global chromatin accessibility changes (**B**). Heatmaps of significantly regulated peaks (signal score > 250; FDR < 0.1), highlighting regions with dynamic accessibility changes in response to vitamin D_3_ (**C**). Pie charts below each heatmap indicate the genomic distribution of the corresponding peaks, categorized as TSS regions, proximal enhancers, and distal enhancers. Manhattan plots display the ATAC-seq signal intensities of consensus peaks for each time point (**D**). The number of significantly enriched peaks (signal score > 250; FDR < 0.1) is annotated for each day.

In a triplicate design of a medical experiment, a high vitamin D responder identified in the VitDHiD trial^55^ received a monthly bolus of 80,000 IU vitamin D_3_ for three consecutive months (Fig. 1A). Blood samples were collected immediately before supplementation (d0), as well as one (d1) and two (d2) days after each dose. The average 25(OH)D_3_ serum level at d0 was 39.8 ng/ml, which increased by 7.5 ng/ml at d1 and by further 6.6 ng/ml at d2. In both instances, vitamin D status declined back to baseline before the subsequent bolus was administered. PBMCs were isolated directly after blood collection for epigenome-wide analysis using ATAC-seq and transcriptome-wide profiling via RNA-seq.

ATAC-seq analysis of all nine samples identified a total of 259,664 peaks, of which 33,836 accessible chromatin regions were consistently detected across all conditions (Table S2). PCA of these consensus peaks revealed the effects of vitamin D_3_ supplementation at d1 and d2 (Figure S1A). A clearer pattern emerged from a peak intensity heatmap comparing d0, d1, and d2, showing the highest average peak signal at d1 (Fig. 1B). Among the 33,836 accessible regions, 11,084 mapped to promoters (within 500 bp of TSSs), 6,943 to proximal enhancers (0.5–10 kb from TSSs), and 15,809 to distal enhancers (10–500 kb from TSSs). Regions both upstream and downstream of the TSS were considered. Notably, the heatmap of promoter-associated peaks (Figure S2A) demonstrated markedly stronger signals compared to those at enhancer regions (Figure S2B).

Applying the criteria of FDR < 0.1 in both comparisons (d1 versus d0, and d2 versus d0) and an average signal intensity > 250, we identified 3,538 differentially accessible peaks, representing more than 10% of all open chromatin regions (Table S2). In contrast, for the most prominent peaks, baseline levels were consistent across biological replicates, with no significant differences detected. Interestingly, although signal intensities of 3,313 peaks at d1 and 4,206 peaks at d2 were reduced relative to d0, all 3,538 differentially accessible regions exhibited increased accessibility upon vitamin D_3_ supplementation; none showed a loss of accessibility. The corresponding heatmap confirms this trend, with peak intensities highest at d1 and lowest at baseline (Fig. 1C). Notably, the majority of these peaks (2,538) were located at promoters, while 528 and 472 mapped to proximal and distal enhancers, respectively. PCA based on the 3,538 significant chromatin regions reveals a clear separation of d1 and d2 from d0, particularly when considering each biological replicate individually (Figure S1B).

Representative genome browser tracks monitor changes in chromatin accessibility at TSS regions of the genes *HMGCR* (3-hydroxy-3-methylglutaryl-CoA reductase; Figure S3A), *GABPA* (Figure S3B), *GYG1* (glycogenin 1; Figure S3C), and *STOM* (stomatin; Figure S3D). For *GYG1* and *STOM*, vitamin D-sensitive enhancer regions are also shown. HOMER motif analysis of the 2,538 significantly altered TSS regions revealed the top five enriched transcription factor binding sites as those for NFY (nuclear transcription factor Y), SP2 (SP2 transcription factor), ELF1 (E74-like ETS transcription factor 1), CREB (cAMP response element-binding protein), and GFY (general transcription factor Y) (Figure S4). In contrast, motif analysis of the 1,000 significantly regulated enhancer regions identified the top five enriched motifs corresponding to EHF (ETS homologous factor), KLF5 (KLF transcription factor 5), RUNX1, CEBP, and JUN (Jun proto-oncogene, AP-1 transcription factor subunit). Notably, KLF5 and RUNX1 are context-dependent transcription factors that can act either as activators or repressors^63,64^. Their regulatory function is influenced by factors such as post-translational modifications and interactions with specific cofactors.

In contrast, VDR binding motifs were absent from the lists of significantly enriched motifs at both TSS and enhancer regions. Because VDR binding and chromatin engagement occur rapidly after ligand exposure (within minutes to hours), our sampling at d1 and d2 is expected to capture sustained rather than immediate primary VDR effects. The accessibility changes we observe may therefore reflect secondary regulatory responses, rather than direct ligand-driven chromatin remodeling alone.

The epigenome-wide impact of vitamin D_3_ bolus supplementation becomes even more pronounced when visualized using a Manhattan plot, which displays the signal intensities of all 33,836 accessible chromatin regions across days 0, 1, and 2 (Fig. 1D). Under baseline conditions, 3,807 ATAC-seq peaks exceed the signal threshold of 250 and are evenly distributed across the genome. Remarkably, 6908 peaks surpass this threshold on d1, with 6,592 also meeting the FDR threshold of 0.1. On d2, 6,246 peaks still show signal intensities above 250, of which 3,588 remain below the FDR threshold.

In summary, we investigated the epigenomic effects of repeated vitamin D_3_ bolus supplementation in a high responder from the VitDHiD cohort. ATAC-seq analysis of PBMCs across three time points revealed over 3,500 chromatin regions with increased accessibility, particularly at promoters, indicating a strong and consistent epigenomic response to vitamin D_3_. Transcription factor motif enrichment suggested the involvement of key regulatory factors, though not VDR directly, highlighting complex gene regulatory mechanisms in response to vitamin D.

### Transcriptome-wide effects of vitamin D_3_ supplementation

**Fig. 2:**
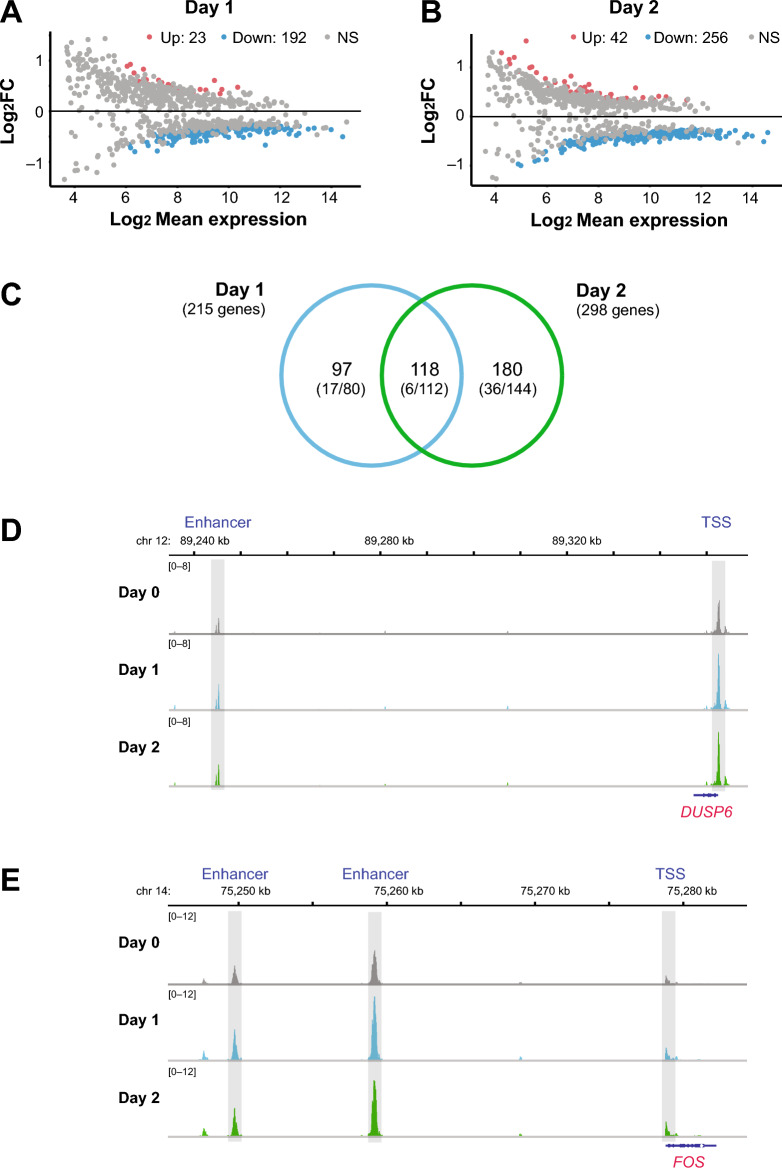
Transcriptional programs induced by vitamin D_3_ supplementation. MA plots showing genes significantly upregulated (red) and downregulated (blue) at day 1 (**A**) and day 2 (**B**) post-supplementation (FDR < 0.05). NS indicates the majority of expressed genes that show no significant response (grey) to vitamin D_3_ supplementation. A Venn diagram illustrates the overlap between differentially expressed genes on days 1 and 2 (**C**). Numbers in parentheses indicate the counts of up- and downregulated genes. ATAC-seq signal profiles were visualized in the IGV browser for the genomic regions surrounding the vitamin D target genes *DUSP6* (**D**) and *FOS* (**E**) across days 0 (grey), 1 (blue), and 2 (green). Vitamin D₃-responsive enhancer and TSS regions are shaded in light grey. Vitamin D target genes are highlighted in red. Tracks show merged data from three biological replicates.

To evaluate the functional consequences of the epigenomic changes induced by vitamin D_3_ supplementation, transcriptome profiling was conducted using RNA-seq on PBMC aliquots from the same proof-of-principle trial that was used for the epigenomic analysis (Fig. 1A). The analysis focused on 11,743 protein-coding genes (Table S4). PCA revealed transcriptome-wide responses as early as d1, with more pronounced changes observed by d2, compared to baseline (d0) (Figure S5A). MA plots further illustrated the extent of differential gene expression. At d1, 23 genes were significantly upregulated and 192 downregulated (FDR < 0.05; Fig. 2A). By d2, the number of differentially expressed genes increased to 42 upregulated and 256 downregulated (Fig. 2B). A total of 118 genes overlapped between both time points, including six genes consistently upregulated across both days (Fig. 2C). In total, 380 vitamin D target genes were identified across both days of analysis (Table S3). Representative examples include *DUSP6* (dual specificity phosphatase 6; Fig. 2D) and *FOS* (Fos proto-oncogene, AP-1 transcription factor subunit; Fig. 2E). Additional examples, such as *HMGCR*, *GABPA*, *GYG1*, and *STOM*, were already presented in Figure S3. When compared with our identically designed cohort study VitDHiD^55^, which identified 452 target genes (FDR < 0.05), 27 genes, including *DUSP6*, were found to be shared and thus already known as in vivo vitamin D targets (Table S4).

To assess physiological consequences for PBMC function, we performed pathway enrichment on the differentially expressed genes (n = 380) using the EnrichR web tool^65,66^ (Table 1). Based on the Reactome pathway database^67^, significant terms (p_adj_ < 0.05) included “innate immune system,” “immune system,” “neutrophil degranulation,” and “metal ion solute carrier transporter,” suggesting involvement in immune-related functions. WikiPathways^68^ further specified these functional associations with terms such as “type II interferon signaling,” “myometrial relaxation and contraction,” and “macrophage-stimulating protein signaling.” These enriched pathways represent functional immune programs modulated in vivo after vitamin D_3_ supplementation, demonstrating that the transcriptional response reflects physiological PBMC activity rather than chromatin remodeling alone.

**Table 1:** Pathways affected by vitamin D_3_ supplementation. Based on EnrichR analysis the databases Reactome and WikiPathways provided the indicated significantly (p_adj_ < 0.05) enriched terms.

Term	Database	Overlap	p_adj_-value
Innate immune system	Reactome	54 of 1,149	0.0000000039
Immune system	Reactome	80 of 2,150	0.0000000976
Neutrophil degranulation	Reactome	39 of 478	0.0000218000
Metal ion solute carrier transporters	Reactome	5 of 24	0.0219383234
Type II interferon signaling (WP619)	WikiPathways	6 of 38	0.0345999197
Myometrial relaxation and contraction pathways (WP289)	WikiPathways	11 of 156	0.0345999197
Macrophage stimulating protein MSP signaling (WP5353)	WikiPathways	9 of 108	0.0345999197

Taken together, transcriptome profiling of PBMCs following vitamin D_3_ supplementation revealed time-dependent gene expression changes, with 380 vitamin D target genes identified across two days, including key immune-related genes such as *DUSP6* and *FOS*. Functional enrichment analysis indicated that these targets are primarily involved in immune system processes, including innate immunity and interferon signaling pathways.

### Vitamin D-regulated chromatin accessibility and gene regulation

**Fig. 3:**
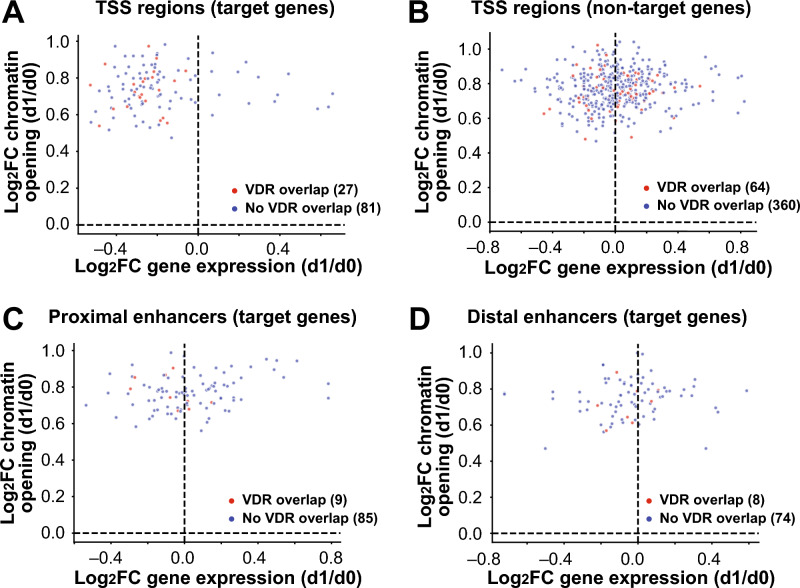
Integration of epigenomic and transcriptomic profiles. The relationship between changes in chromatin accessibility (log_2_FC, shown for d1 versus d0) of 712 vitamin D sensitive chromatin regions and corresponding changes in the expression of 306 target genes is plotted. Data are shown for 108 TSS regions directly associated with vitamin D target genes (**A**), TSS regions (n = 424) linked to non-target genes (**B**), 94 proximal enhancer regions (**C**) and 82 distal enhancers (**D**). Chromatin regions overlapping with a VDR binding site (red) are distinguished from those not known to bind VDR.

To investigate the regulatory contribution of differentially accessible chromatin regions to in vivo vitamin D target genes, we integrated epigenomic (Table S2) and transcriptomic (Table S4) datasets. Of the 3,538 regions showing differential accessibility, 3,093 were located near protein-coding genes. Among these, 712 lay within 500 kb of a vitamin D target gene, including 108 at the TSS of target genes and 604 at other regulatory sites, specifically, 82 distal enhancers, 94 proximal enhancers, and 428 TSS regions of non-target genes that may act as enhancers (Fig. 3).

Functional relevance of these regions was supported by VDR ChIP-seq data obtained from THP-1 cells^30^, revealing that 2,319 (6.7%) of all 33,836 accessible chromatin regions overlapped known VDR binding sites. Of the 3,093 differentially accessible regions near protein coding genes, 490 (15.8%) overlapped with VDR binding, including 381 TSSs, 61 proximal enhancers, and 48 distal enhancers. Among these, 108 (91 TSSs, 9 proximal enhancers, 8 distal enhancers) were within 500 kb of vitamin D target genes (red dots in Fig. 3A-D). VDR binding was more frequent at TSSs (17.0%; Fig. 3A,B) than enhancers (9.7%; Fig. 3C,D) and more common at TSSs of target genes (25.0%; Fig. 3A) than non-target genes (15.1%; Fig. 3B). Although only 14 of the 108 target genes with vitamin D-sensitive TSSs showed transcriptional upregulation after vitamin D_3_ supplementation, all TSS regions displayed increased chromatin accessibility. A similar trend was observed at TSSs of non-target genes and at enhancer regions.

Of the 380 in vivo vitamin D target genes, 306 were associated with at least one vitamin D-sensitive chromatin region within 500 kb of the TSS (Table S4). Among these, 122 were linked via TSS regions and 184 via one or more enhancer elements, many of which overlapped with TSSs of non-target genes, suggesting long-range regulation. Some genes, such as *GAPDH* (glyceraldehyde-3-phosphate dehydrogenase) and *SRCAP* (Snf2-related CREBBP activator protein), were associated with as many as 14 vitamin D-sensitive enhancer regions. In total, up to 935 distinct TSS and enhancer regions appear to be involved in regulating these 306 target genes.

Further analysis revealed redundancies in regulatory element counts due to the close proximity of target genes. Examples of such genomic constellations include *CCNG1* (cyclin G1)/ *MAT2B* (methionine adenosyltransferase 2β), *SERPINB1* (serpin family B member 1)/ *SERPINB3*, and *KLF10*/ *AZIN1* (antizyme inhibitor 1), where shared enhancers or overlapping TSSs suggest coordinated regulation within clustered gene regions (Fig. S6A–C). Both *CCNG1* and *MAT2B* harbor vitamin D-sensitive TSS regions and appear to be co-regulated by two nearby enhancers, one of which also functions as the TSS of the *HMMR* (hyaluronan-mediated motility receptor) gene. The *SERPINB1*/*SERPINB9* gene pair is regulated by four enhancers, two of which also serve as promoters. Likewise, *KLF10* and *AZIN1* are regulated by a single enhancer and the TSS of the *GASAL1* (growth arrest-associated lncRNA 1) gene.

In summary, integration of epigenomic and transcriptomic data revealed that 306 of 380 in vivo vitamin D target genes are associated with nearby vitamin D-sensitive chromatin regions, including both TSSs and enhancers, many of which overlap with VDR binding sites and TSSs of non-target genes, suggesting long-range regulatory interactions. Redundancy in regulatory elements was observed due to gene proximity, with shared enhancers and TSSs co-regulating neighboring genes.

### Epigenome-wide vitamin D response of a cohort

**Fig. 4:**
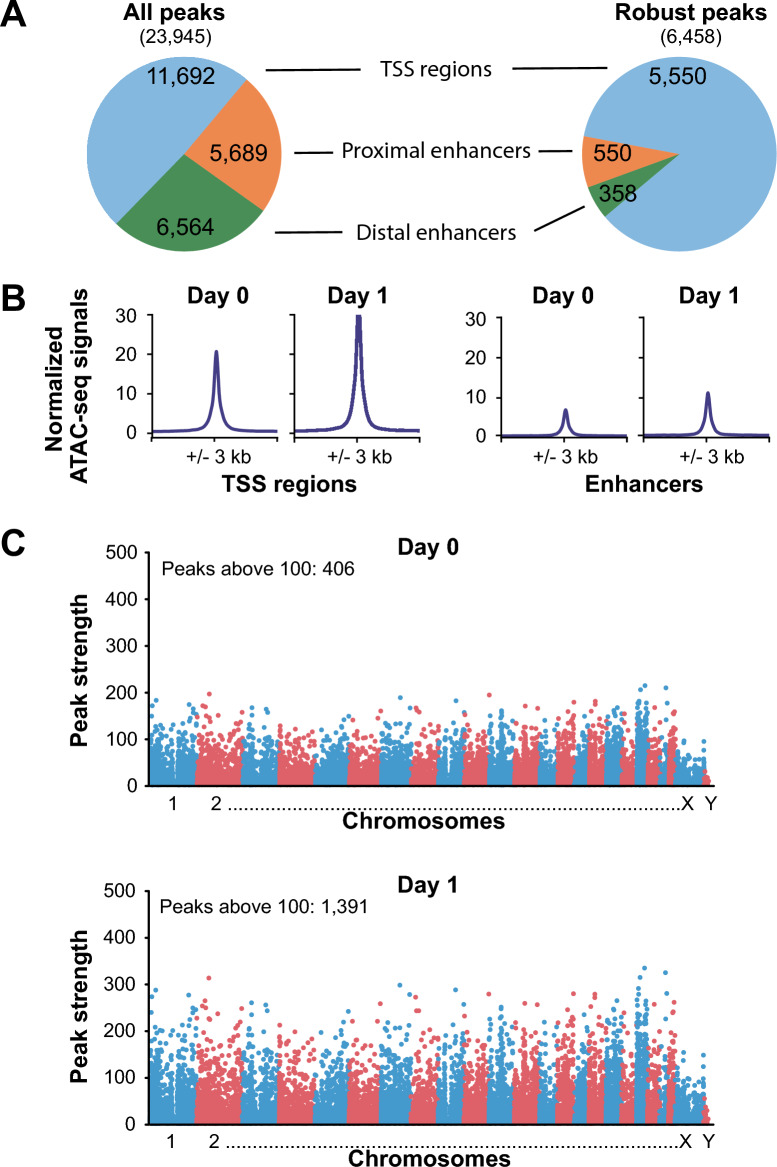
Vitamin D-induced chromatin accessibility of the cohort. Pie charts indicate the genomic distribution of the all 23,945 consensus peaks (left) and the 6,458 robust peaks (right), categorized as TSS regions, proximal enhancers, and distal enhancers (**A**). ATAC-seq signal intensities of all TSS regions (left) and enhancer regions (right) at d0 and d1 illustrate global chromatin accessibility changes (**B**). Manhattan plots of ATAC-seq signal intensities for all consensus peaks at d0 and d1 (**C**). The number of significantly enriched peaks (signal score > 100) is indicated for each time point.

A cohort of 13 individuals from the vitamin D intervention study VitDPAS was selected to evaluate inter-individual variation in the PBMC epigenome’s response to vitamin D_3_ supplementation. As in the VitDHiD trial^55^, participants received a vitamin D_3_ bolus (1,000 IU/kg), but in this case it was administered only once, with blood samples collected at d0 and d1. The mean 25(OH)D_3_ serum concentration increased from 25.7 ng/ml at d0 to 31.6 ng/ml at d1^56^. ATAC-seq analysis identified 23,945 consensus peaks across all 26 samples (Table S3), with 11,692 mapping to TSS regions, 5,689 to proximal enhancers, and 6,564 to distal enhancers (Fig. 4A). Focusing on the 6,458 robust peaks (CPM > 10), the majority were located in TSS regions (86.0%), with only 8.5% in proximal enhancers and 5.5% in distal enhancers. This distribution is consistent with the heatmap of promoter-associated peaks, which shows markedly stronger signals compared with those at enhancer regions (Fig. 4B). At d0, only 406 peaks showed an average signal intensity > 100, whereas at d1 this number increased to 1,391 (Fig. 4C), indicating a pronounced gain in chromatin accessibility, consistent with the N-of-1 approach (Fig. 1D).

**Fig. 5:**
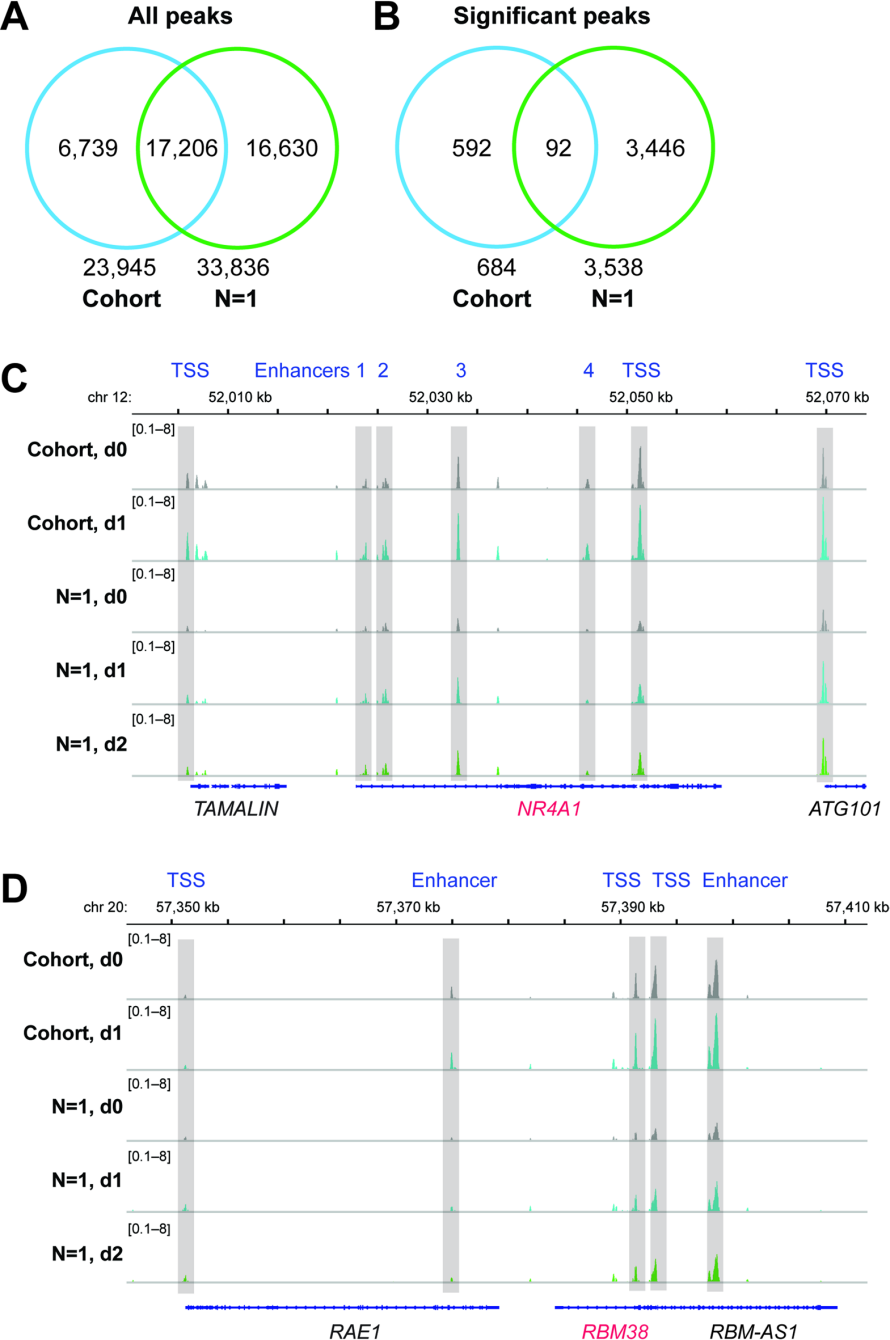
Comparing cohort and N-of-1 approaches. Venn diagrams compare the cohort and N-of-1 approach for all consensus peaks (**A**) and significantly regulated peaks (**B**). Representative genomic regions of vitamin D–responsive chromatin accessibility for the target genes *NR4A1* (**C**) and *RBM38* (**D**), visualized in the IGV browser. Enhancer and TSS regions are shaded in light grey, with vitamin D target genes highlighted in red. Tracks display merged ATAC-seq data from 13 individuals in the VitDPAS trial (d0, d1) and from three biological replicates in the N-of-1 approach (d0, d1, d2).

A direct comparison of the cohort and N-of-1 approaches revealed that 71.9% of the consensus peaks were identical (Fig. 5A), leaving 16,630 peaks unique to the individual from the VitDHiD trial. In the cohort approach, only 684 peaks were significantly (p < 0.05) regulated by vitamin D_3_ supplementation (Table S3), of which 92 had already been observed in the N-of-1 approach (Fig. 5B). Notably, this means that the vast majority (97.4%) of significant peaks identified in the N-of-1 design with three biological replicates could not be detected using the cohort approach. Nevertheless, representative genome browser tracks illustrate matching regions of chromatin accessibility and their regulation in both approaches, as exemplified by the vitamin D target genes *NR4A1* (nuclear receptor subfamily 4 group A member 1; Fig. 5C) and *RBM38* (RNA-binding motif protein 38; Fig. 5D).

Taken together, in the VitDPAS cohort of 13 individuals receiving a single vitamin D_3_ bolus, ATAC-seq revealed a pronounced overall gain in promoter-associated chromatin accessibility from d0 to d1, with changes largely consistent with findings from the N-of-1 approach. However, the cohort approach detected far fewer significantly regulated peaks than the N-of-1 design, with only 2.6% overlap in significant sites, despite matching regulation at exemplar vitamin D target loci such as *NR4A1* and *RBM38*.

## Discussion

This study presents a comprehensive in vivo analysis of the epigenomic and transcriptomic effects of high-dose vitamin D_3_ supplementation in a healthy individual classified as a high vitamin D responder based on the vitamin D response index^55^. A high responder was chosen because such individuals display more pronounced gene expression changes despite only modest increases (~ 35%) in 25(OH)D_3_ serum levels two days post-supplementation. Integration of ATAC-seq and RNA-seq data from PBMCs collected over three consecutive bolus applications revealed a robust, consistent genome-wide epigenetic response to vitamin D_3_, accompanied by dynamic gene expression changes predominantly associated with immune regulation.

The most striking epigenomic finding was a genome-wide increase in chromatin accessibility, especially at promoter regions, after a single vitamin D_3_ bolus (80,000 IU). Notably, none of the 3,538 differentially accessible regions showed reduced accessibility. Despite this global chromatin opening, most of the 380 identified vitamin D target genes were downregulated. This pattern may reflect feedback regulation or anti-inflammatory effects, consistent with vitamin D’s role in modulating signal transduction pathways that help maintain systemic homeostasis^69^. The observed disconnect between chromatin accessibility changes and gene expression implies that chromatin opening may serve to prime genes for later inactivation or to enable alternative regulatory processes, such as enhancer RNA transcription or noncoding RNA production^70^. Although the strongest accessibility changes occurred at TSS regions, both proximal and distal enhancers also contributed to the vitamin D response. Notably, many promoters of transcriptionally unregulated genes showed increased chromatin accessibility, suggesting they may act as enhancers for vitamin D target genes rather than as promoters in this context.

Motif enrichment analysis indicated a predominance of short binding motifs for pioneer and general transcription factors, such as members of the NFY, KLF, RUNX, and ETS families, rather than the more complex motifs typically associated with VDR–RXR heterodimers. The lack of significant enrichment for canonical VDR motifs among regulated regions suggests indirect regulatory mechanisms, including cooperative interactions with the identified pioneer factor motifs. Nevertheless, over 15% of differentially accessible chromatin regions overlapped with VDR ChIP-seq peaks previously identified in THP-1 cells^29,30^, providing experimental evidence that some regions directly bind VDR.

Consistent with our findings, even in vitro studies have shown that only 15–20% of VDR ChIP-seq peaks contain the canonical DR3-type binding motif^29^, indicating that its absence in our analysis aligns with previous observations of VDR–RXR heterodimer binding. Transcriptome-wide analysis identified 380 protein-coding genes differentially expressed after vitamin D_3_ supplementation, including well-established targets such as *DUSP6*^71^*, FOS*^72^, and *SERPINB1*^69^, all known to participate in feedback regulation of inflammation^73,74^. Taken together, these observations support a multi-stage regulatory model in which initial VDR activation is followed by secondary transcription factor networks and cytokine signaling cascades that shape the immune transcriptional response.

Overall, vitamin D_3_ supplementation was associated with a predominant downregulation of gene expression, consistent with previous in vivo and in vitro studies reporting more down- than upregulated genes after ≥ 24 h of vitamin D_3_ or 1,25(OH)_2_D_3_ exposure^9,55,75^. Transcriptomic analysis indicated that vitamin D–responsive genes were significantly enriched in pathways related to innate immunity, interferon signaling, and macrophage activation. This is in line with clinical and epidemiological evidence linking vitamin D status to immune competence and inflammation control^4,76^.

Importantly, several differentially expressed genes identified in our study, including *DUSP6*, *FOS*, and AP-1 components, have also been reported as vitamin D–responsive in independent cohorts such as VitDHiD^55^, supporting the reproducibility and biological robustness of these regulatory effects. This cross-cohort consistency provides validation complementary to traditional qPCR-based confirmation approaches.

Of the 380 differentially expressed genes detected here, 27 overlapped with the 452 vitamin D target genes reported in VitDHiD, reflecting substantial inter-individual variability in transcriptional responses to vitamin D^57,77^. This variability emphasizes the context- and cell-type-specific nature of vitamin D signaling, which likely contributes to its wide range of physiological and pathological roles^78^.

Integration of ATAC-seq and RNA-seq data yielded deeper mechanistic insights. Many of these regions overlapped with known VDR binding sites, particularly at target gene TSSs, suggesting that vitamin D-induced chromatin accessibility changes directly contribute to transcriptional regulation. Moreover, VDR binding was detected at enhancer regions and at promoters of non-target genes, suggesting a complex regulatory network that includes long-range chromatin interactions and coordinated regulation of gene clusters^79^. Examples such as the genomic loci of *CCNG1*/*MAT2B*, *KLF10*/*AZIN1*, and *SERPINB1*/*SERPINB9* illustrate how vitamin D-sensitive enhancers may coordinate gene expression across broader genomic neighborhoods^41^.

Despite these pronounced molecular responses, only a subset of accessible chromatin regions and VDR-bound sites produced detectable transcriptional changes. This supports the idea that chromatin accessibility is permissive but not, on its own, sufficient for transcriptional activation^80^. Instead, it creates the potential for responsiveness under appropriate cellular conditions or in the presence of additional stimuli. The complex, multi-layered nature of vitamin D-mediated gene regulation warrants further investigation, ideally using high-resolution time-course studies and integrating proteomic and metabolomic data to better capture downstream functional effects.

The VitDPAS cohort analysis serves as an important link between the highly controlled, repeated-measures N-of-1 approach and the complexity of population-level responses to vitamin D_3_ supplementation. Although both approaches revealed a global gain in promoter-associated chromatin accessibility within 24 h of a bolus, the magnitude and statistical detectability of these changes were markedly lower in the cohort. Only 684 regions reached nominal significance in the VitDPAS dataset, compared with 3,538 in the high-responder N-of-1 study, with 97.4% of N-of-1-specific significant peaks absent from the cohort results. This difference is likely due to a combination of biological heterogeneity, differences in baseline vitamin D status, genetic and epigenetic backgrounds, and variations in immune cell composition among participants. Such inter-individual variability, repeatedly noted in vitamin D research^27,57,77^, supports the concept of the personal vitamin D response index.

The limited overlap of significant peaks between the N-of-1 and cohort datasets should not be seen as contradicting the single-subject findings, but rather as illustrating the statistical dilution that occurs when pooling data from individuals with asynchronous or weaker responses. The comparison between the two designs also emphasizes an important methodological consideration for nutrigenomic and epigenomic intervention studies: while large cohort designs capture population-level trends, they may underestimate the magnitude and scope of molecular responses detectable in within-subject, repeated-measures experiments. This is particularly relevant for interventions such as vitamin D_3_ supplementation, where both response kinetics and magnitude vary greatly between individuals. A hybrid strategy that combines the statistical generalizability of cohort studies with the sensitivity of a longitudinal N-of-1 design may therefore provide the most complete view of nutrient-driven molecular remodeling. In the context of vitamin D, such an approach could clarify both the personalized and shared regulatory architecture of its action, offering a stronger basis for precision supplementation strategies.

From a clinical and translational standpoint, our findings show that vitamin D_3_ supplementation can induce favorable epigenetic modifications^81^, although the magnitude of these changes varies greatly among individuals. High responders display robust and specific molecular effects, supporting the concept of personalized supplementation^27^, whereas many individuals exhibit measurable but statistically less pronounced changes, suggesting only partial engagement of vitamin D–sensitive regulatory pathways. These observations emphasize the need to account for inter-individual variability in both mechanistic studies and intervention trials, with tools such as the vitamin D response index serving as potential molecular biomarkers to guide tailored dosing strategies and optimize immune health and disease prevention^82^.

In addition to classical genomic signaling through VDR in the nucleus, vitamin D metabolites can also induce rapid non-genomic signaling responses^83^, including activation of intracellular kinase cascades and second messenger systems. Such non-genomic pathways have been proposed to modulate chromatin and transcription indirectly, for example by regulating the activity or nuclear availability of downstream transcription factors. Therefore, it is possible that some of the differential gene expression patterns observed in our study represent the integrated outcome of both direct VDR-mediated transcriptional regulation and secondary effects mediated by non-genomic signaling. This layered regulatory architecture aligns with the multi-stage immune modulation that we observe in PBMCs following vitamin D_3_ supplementation.

This study has several limitations. Most importantly, its core component focuses on a single high responder, which restricts the generalizability of the results. To mitigate this limitation, the cohort approach included 4 high, 5 mid, and 4 low responders from the VitDPAS study. Second, both vitamin D intervention trials used in the context of this study had a non-randomized design. Third, the analysis was conducted on PBMCs, which represent a mixture of innate and adaptive immune cells. The dominant pathways we observe reflect immune signaling programs, while different tissues and immune cell subsets may exhibit distinct temporal and regulatory responses to vitamin D. Fourth, although the repeated-measures N-of-1 design provides high sensitivity, it is inherently limited in scope and cannot represent the full range of population variability. Finally, while our integrated ATAC-seq and RNA-seq analysis offers valuable mechanistic insights, functional validation of specific regulatory elements and target genes will be essential to confirm their roles in vitamin D_3_–mediated immune modulation.

In conclusion, this study shows that high-dose vitamin D_3_ supplementation triggers rapid, widespread chromatin remodeling in PBMCs, accompanied by coordinated transcriptional changes in immune-related pathways. The combination of high-resolution N-of-1 and cohort-based designs offers complementary perspectives on the dynamics and variability of the vitamin D response, revealing both shared and individualized regulatory programs. These findings emphasize the potential of personalized vitamin D supplementation strategies, guided by molecular profiling, to optimize immune health and disease prevention.

## Supplementary Information

Supplementary Information 1.

Supplementary Information 2.

Supplementary Information 3.

Supplementary Information 4.

Supplementary Information 5.

Supplementary Information 6.

Supplementary Information 7.

Supplementary Information 8.

Supplementary Information 9.

Supplementary Information 10.

Supplementary Information 11.

Supplementary Information 12.

Supplementary Information 13.

Supplementary Information 14.

## Data Availability

The raw sequencing data (FASTQ files) are available at the Gene Expression Omnibus (GEO, www.ncbi.nlm.nih.gov/geo/) under accession numbers GSE295550 (ATAC-seq, N-of-1 approach), GSE303320 (ATAC-seq, cohort approach) and GSE295549 (RNA-seq, N-of-1 approach).

## Abbreviations

1,25(OH)_2_D_3_: 1α,25-Dihydroxyvitamin D_3_
25(OH)D_3_: 25-Hydroxyvitamin D_3_
ATAC-seq: Assay for transposase-accessible chromatin with high-throughput sequencing
AZIN1: Antizyme inhibitor 1
BACH2: BTB domain and CNC homolog 2
CCNG1: Cyclin G1
CEBP: CCAAT enhancer binding protein
ChIP-seq: Chromatin immunoprecipitation sequencing
CPM: Counts per million
CREB: CAMP responsive element binding protein
DNase-seq: Dnase I hypersensitivity sequencing
DUSP6: Dual specificity phosphatase 6
EHF: ETS homologous factor
ELF1: E74 like ETS transcription factor 1
ETS1: ETS proto-oncogene 1, transcription factor
FAIRE-seq: Formaldehyde-assisted isolation of regulatory elements sequencing
FC: Fold change
FDR: False discovery rate
FOS: Fos proto-oncogene, AP-1 transcription factor subunit
GABPA: GA binding protein transcription factor subunit alpha
GAPDH: Glyceraldehyde-3-phosphate dehydrogenase
GASAL1: Growth arrest associated LncRNA 1
GFY: General transcription factor Y
GYG1: Glycogenin 1
HMGCR: 3-Hydroxy-3-methylglutaryl-CoA reductase
HMMR: Hyaluronan mediated motility receptor
HOMER: Hypergeometric optimization of motif EnRichment
IGV: Integrative genomics viewer
JUN: Jun proto-oncogene, AP-1 transcription factor subunit
KLF5: KLF transcription factor 5
MAT2B: Methionine adenosyltransferase 2 non-catalytic beta subunit
NFY: Nuclear transcription factor Y
NR4A1: Nuclear receptor subfamily 4 group A member 1
PBMC: Peripheral blood mononuclear cell
PCA: Principal component analysis
PU.1: Purine-rich box-1
RBM38: RNA binding motif protein 38
RNA-seq: RNA sequencing
RUNX: RUNX family transcription factor
RXR: Retinoid X receptor
SERPINB: Serpin family B member
SP2: SP2 transcription factor
SRCAP: Snf2 related CREBBP activator protein
STOM: Stomatin
TAD: Topologically associating domain
TSS: Transcription start site
VDR: Vitamin D receptor
