# Supplementary material for: Chromatin and transcriptional dynamics underlying the immune-modulatory effects of vitamin D3 in vivo
Source: Sci Rep. 2025 Dec 18;16:2997. doi: 10.1038/s41598-025-32831-z (PMC12830676; doi:10.1038/s41598-025-32831-z)
Supplement: Supplementary file 1 — Supplementary Information 1. [file 41598_2025_32831_MOESM1_ESM.pdf]

CARE Checklist – VitDHiD (N-of-1 Intervention)

Title:

✓ Clearly indicates this is an N-of-1, repeated-measures interventional study.

Abstract:

✓ Summarizes participant, intervention, and outcome measurements.

Introduction:

✓ Rationale for assessing high vitamin D responder in a controlled repeated-measures setting.

Participant Information:

- Single healthy male, age 59, high vitamin D responder.
- Baseline 25(OH)D level ~40 ng/mL.
- Informed consent obtained.

Clinical Findings:

✓ No clinical symptoms; study aimed at molecular profiling only.

Timeline:

- **Day 0 (d0):** Baseline blood collection.
- **Day 1 (d1):** 24 hours post-bolus.
- **Day 2 (d2):** 48 hours post-bolus.
- Repeated for three consecutive months.

Diagnostic Assessment:

✓ None; standard molecular assays (ATAC-seq, RNA-seq).

Therapeutic Intervention:

- Monthly oral bolus of **80,000 IU vitamin D_3** for three months.

Follow-up and Outcomes:

- Epigenomic and transcriptomic profiling at each time point.
- No adverse events observed.

Discussion:

- Benefits: High-resolution mechanistic insight.
- Limitations: N=1 design limits generalizability.

Patient Perspective:

- Not applicable for molecular profiling-only study.

Informed Consent:

✓ Written informed consent obtained.
