# Supplementary figures and images for: Chromatin and transcriptional dynamics underlying the immune-modulatory effects of vitamin D3 in vivo
Source: Sci Rep. 2025 Dec 18;16:2997. doi: 10.1038/s41598-025-32831-z (PMC12830676; doi:10.1038/s41598-025-32831-z)

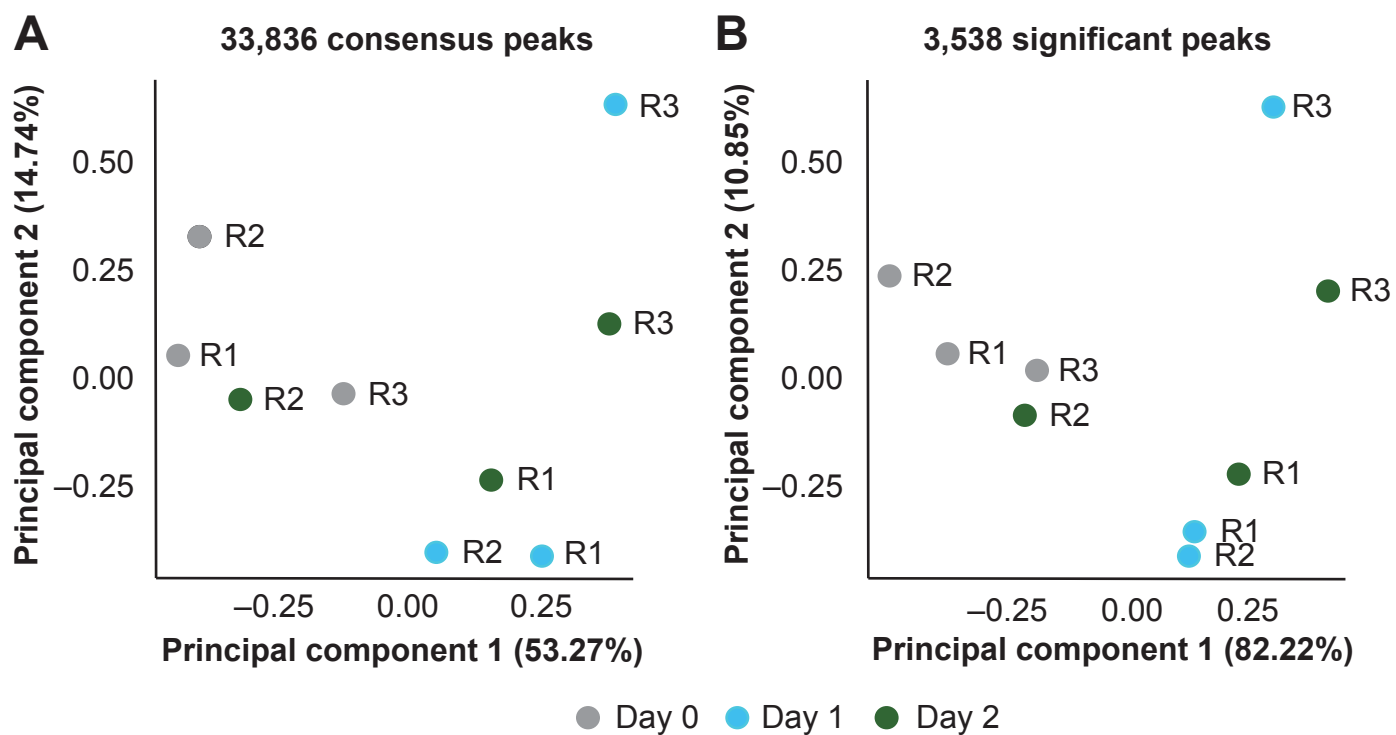

Supplement: Supplementary file 2 — Supplementary Information 2. [file 41598_2025_32831_MOESM2_ESM.pdf]

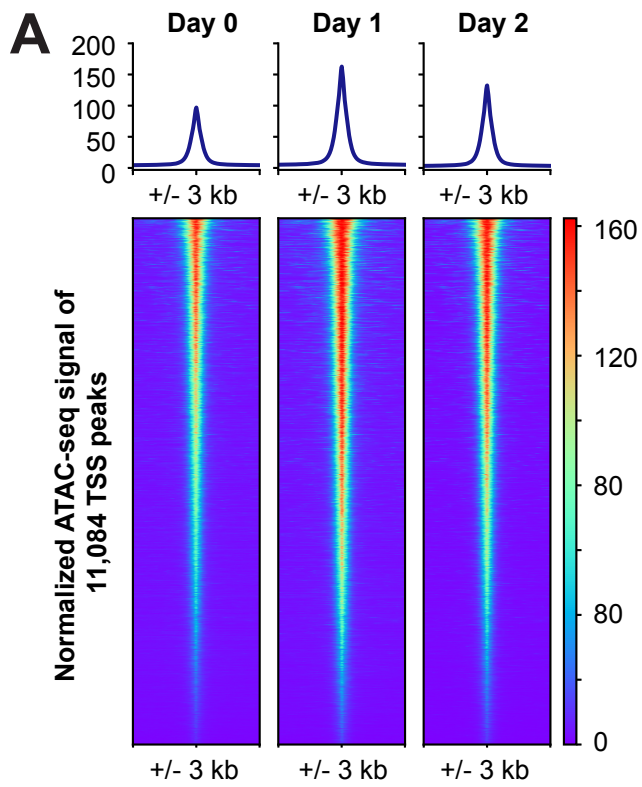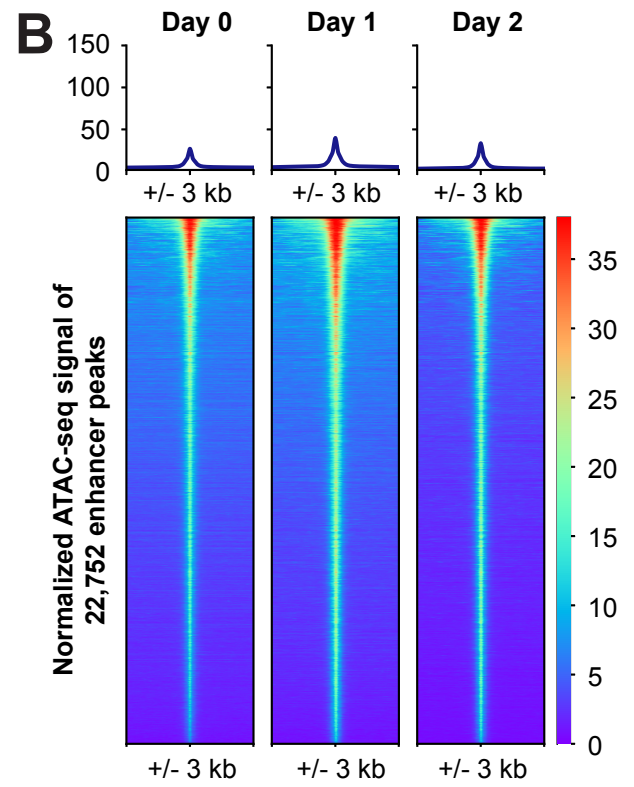

Supplement: Supplementary file 3 — Supplementary Information 3. [file 41598_2025_32831_MOESM3_ESM.pdf]

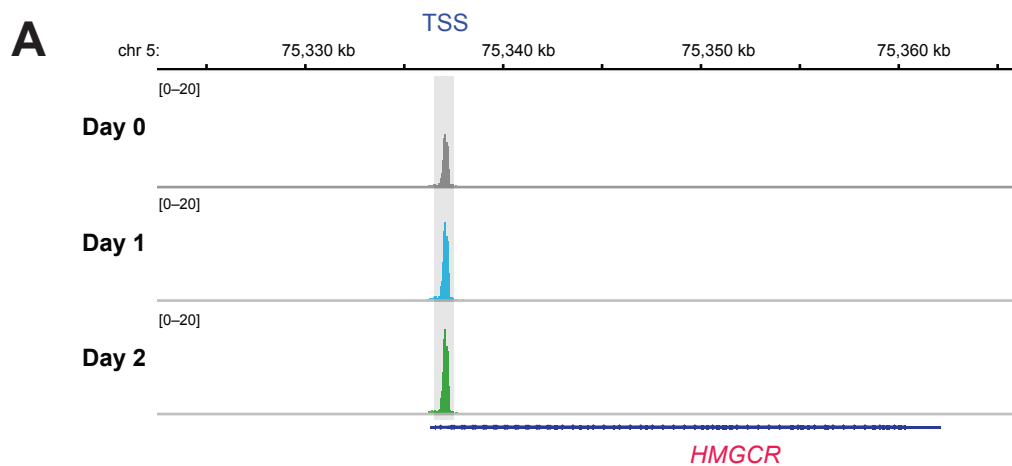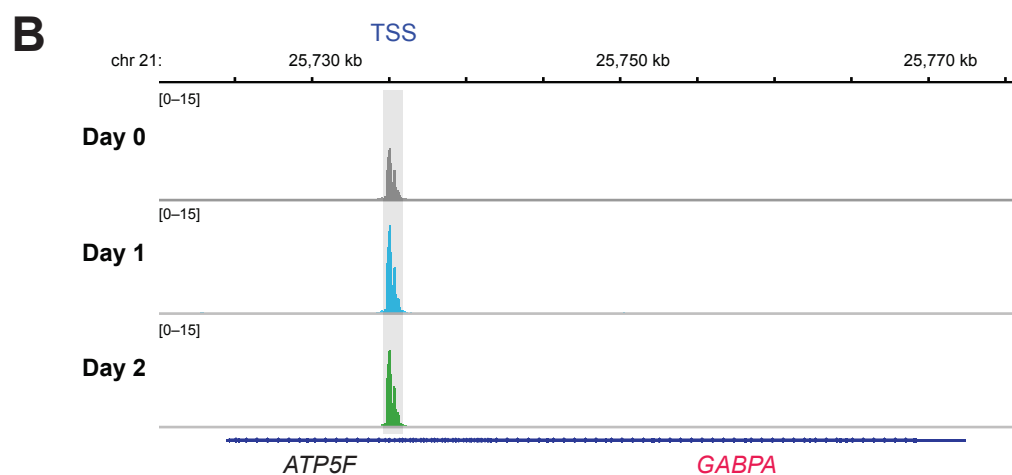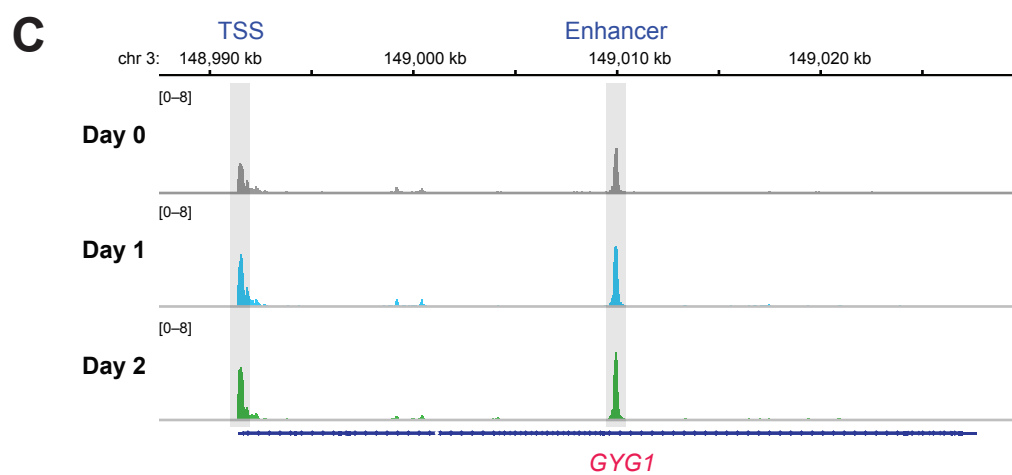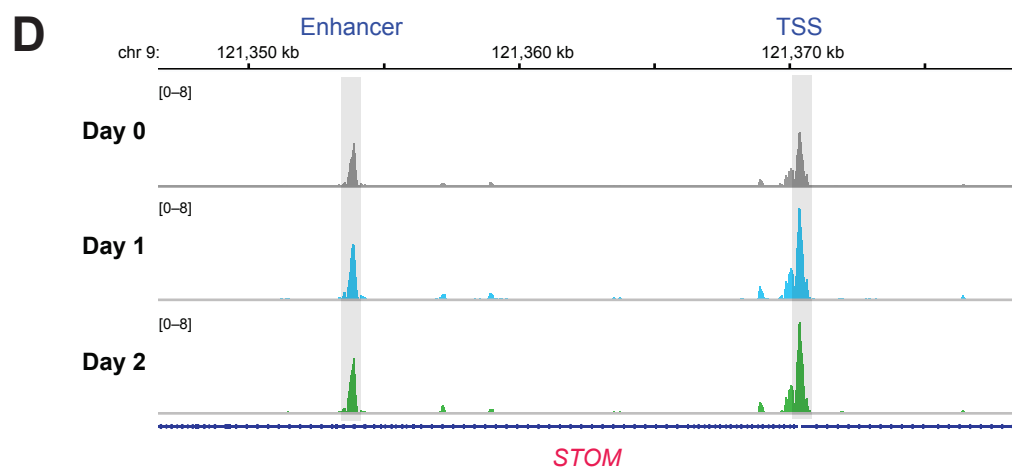

Supplement: Supplementary file 4 — Supplementary Information 4. [file 41598_2025_32831_MOESM4_ESM.pdf]

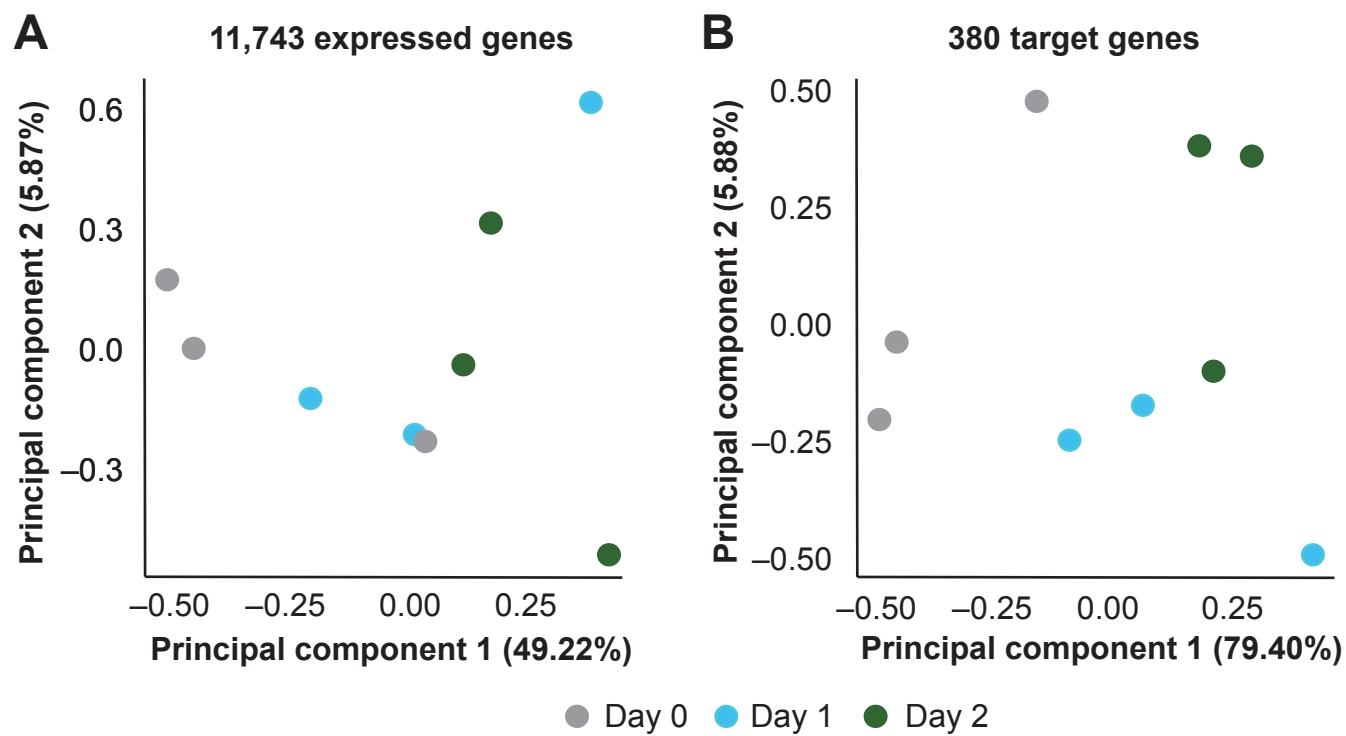

Supplement: Supplementary file 6 — Supplementary Information 6. [file 41598_2025_32831_MOESM6_ESM.pdf]

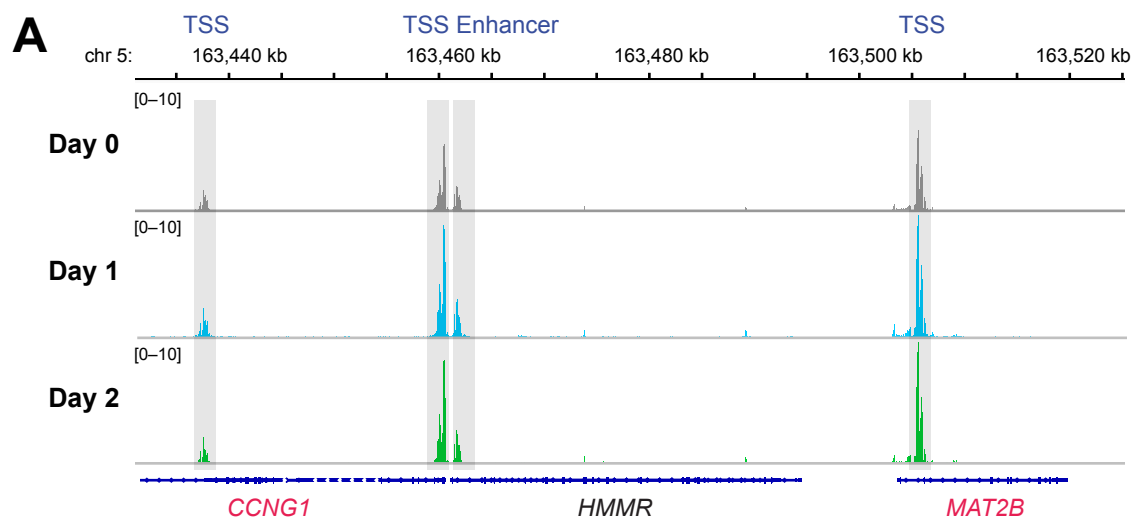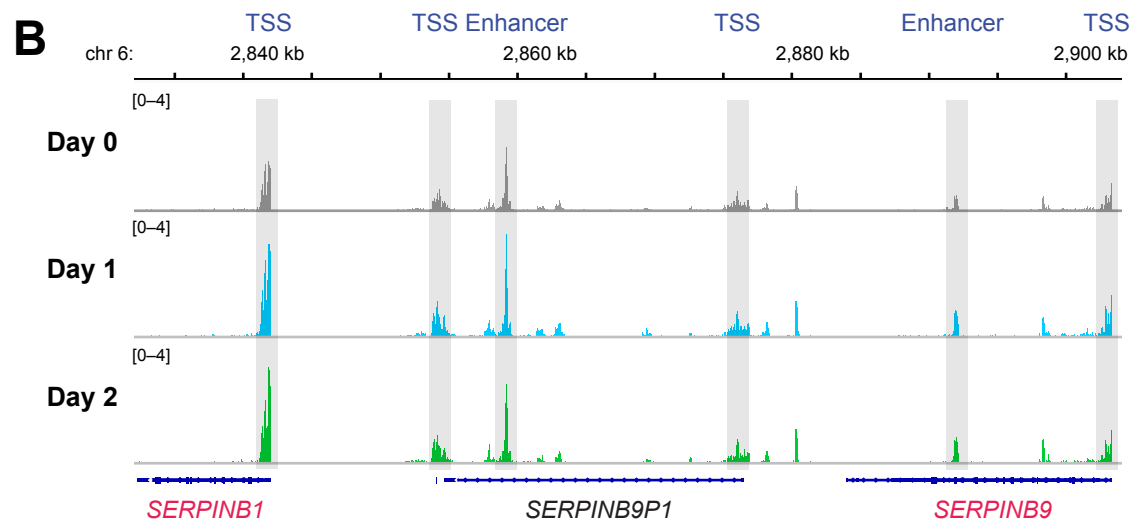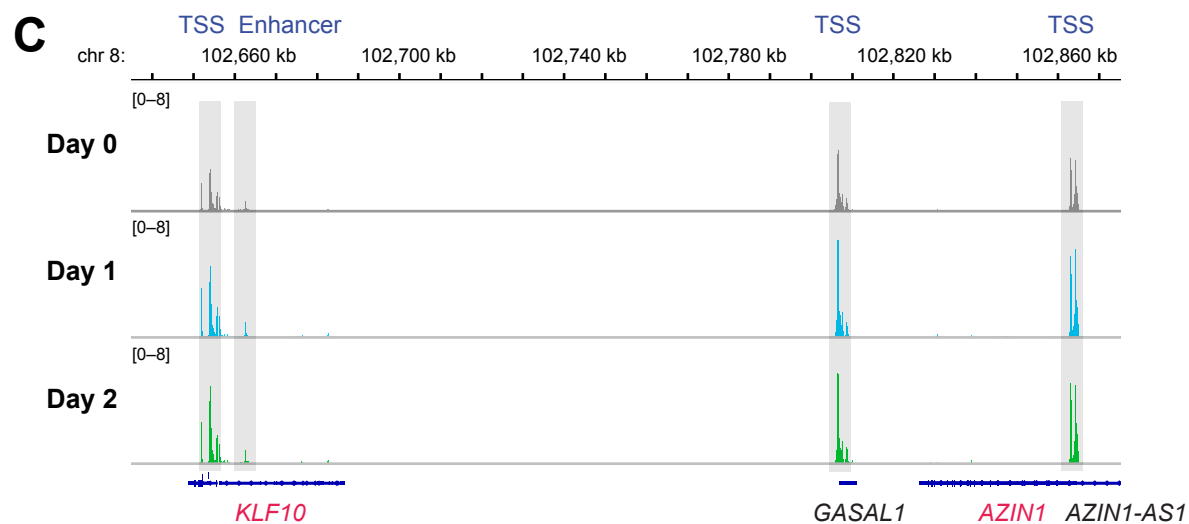

Supplement: Supplementary file 7 — Supplementary Information 7. [file 41598_2025_32831_MOESM7_ESM.pdf]
