# Supplementary material for: Chromatin and transcriptional dynamics underlying the immune-modulatory effects of vitamin D3 in vivo
Source: Sci Rep. 2025 Dec 18;16:2997. doi: 10.1038/s41598-025-32831-z (PMC12830676; doi:10.1038/s41598-025-32831-z)
Supplement: Supplementary file 5 — Supplementary Information 5. [file 41598_2025_32831_MOESM5_ESM.pdf]

	Motif sequence	Transcription factor	p-value	% of sequence
Top 5 of 2,538 TSS regions	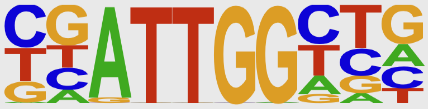	NFY	10^{-243}	17.81%
	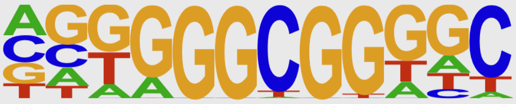	SP2	10^{-128}	23.29%
	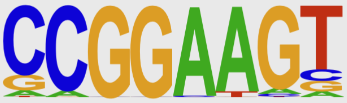	ELF1	10^{-74}	12.96%
	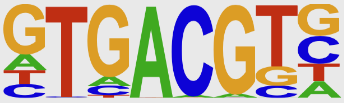	CREB	10^{-42}	11.47%
	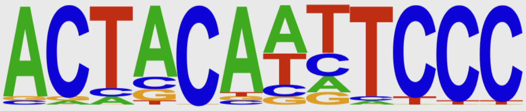	GFY	10^{-36}	2.25%
Top 5 of 1,000 enhancer regions	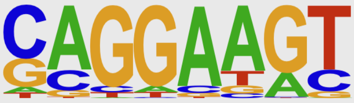	EHF	10^{-58}	17.00%
	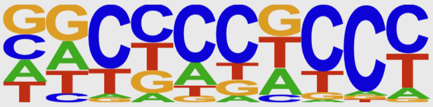	KLF5	10^{-33}	20.10%
	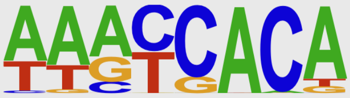	RUNX1	10^{-30}	17.00%
	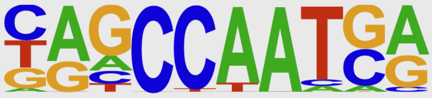	CEBP	10^{-28}	5.90%
	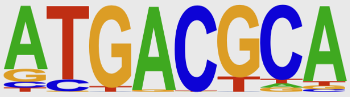	JUN-JUNB	10^{-17}	7.60%
