# Supplementary material for: Chromatin and transcriptional dynamics underlying the immune-modulatory effects of vitamin D3 in vivo
Source: Sci Rep. 2025 Dec 18;16:2997. doi: 10.1038/s41598-025-32831-z (PMC12830676; doi:10.1038/s41598-025-32831-z)
Supplement: Supplementary file 8 — Supplementary Information 8. [file 41598_2025_32831_MOESM8_ESM.pdf]

SUPPLEMENTARY DATA

SUPPLEMENTARY TABLES

Table S1. Read Alignment Statistics. The number of uniquely aligned reads per sample is provided for both ATAC-seq and RNA-seq datasets.

Table S2. Epigenomic Changes in PBMCs (N-of-1 Approach). PBMCs were isolated at d0, d1, and d2 after vitamin D_3 bolus supplementation. ATAC-seq analysis identified 33,836 accessible chromatin regions, of which 3,538 showed significant changes in response to at least one timepoint (highlighted in green).

Table S3. Epigenomic Changes in PBMCs (Cohort Approach). PBMCs were isolated at d0, and d1 after vitamin D_3 bolus supplementation. ATAC-seq analysis identified 23,945 accessible chromatin regions, of which 684 showed significant changes in response to vitamin D (highlighted in green).

Table S4. Transcriptomic Changes in PBMCs (N-of-1 Approach). The same experimental setup as in **Table S2** was applied. RNA-seq analysis identified 11,743 expressed protein-coding genes (CPM > 10), of which 380 responded significantly at at least one time point (highlighted in green).

SUPPLEMENTARY FIGURES

Figure S1. ATAC-seq Sample Quality Control. PCA was performed to evaluate the clustering and reproducibility of ATAC-seq data across the three biological replicates (R1-R3) of the *in vivo* experiment. PCA based on all consensus peaks (**A**) is compared to PCA restricted to significantly regulated peaks (signal score > 250; FDR < 0.1) (**B**). Samples from baseline (day 0) are compared with those from day 1 and day 2 post–vitamin D_3 supplementation, highlighting the temporal effect on chromatin accessibility.

Figure S2. Comparing of Chromatin Accessibility at TSS Regions and Enhancers. Heatmaps showing ATAC-seq signal intensities for all TSS regions (**A**) and enhancers (**B**) across days 0, 1, and 2 post-supplementation, illustrating global chromatin accessibility changes.

Figure S3. Representative Differential Chromatin Accessibility Regions. ATAC-seq signal profiles visualized in the IGV browser for the genomic regions surrounding the vitamin D target genes *HMGCR* (**A**), *GABPA* (**B**), *GYG1* (**C**), and *STOM* (**D**) across days 0 (grey), 1 (blue), and 2 (green). Vitamin D_3-responsive enhancer and TSS regions are shaded in light grey. Vitamin D target genes are highlighted in red. Tracks show merged data from three biological replicates.

Figure S4. Transcription Factor Binding Motif Enrichment. Motif enrichment analysis was performed using HOMER on two sets of regions: 2,538 significantly regulated TSS regions (top panel) and 1,000 vitamin D_3-responsive enhancer regions (bottom panel). The top five enriched transcription factor binding motifs are shown for each set, ranked by p-value.

Figure S5. RNA-seq Sample Quality Control. PCA was performed to evaluate the clustering and reproducibility of RNA-seq data across the three biological replicates of

the *in vivo* experiment. PCA based on all expressed genes (**A**) is compared to PCA restricted to significantly regulated genes (FDR < 0.05) (**B**). Samples from baseline (day 0) are compared with those from day 1 and day 2 post–vitamin D_3 supplementation, highlighting the temporal effect on gene expression.

Figure S6. Examples of Clusters of Differential Chromatin Accessibility Regions. ATAC-seq signal profiles visualized in the IGV browser for the genomic regions surrounding the vitamin D target genes *CCNG1*/*MAT2B* (**A**), *SERPINB1*/*SERPINB9* (**B**), and *KLF10*/*AZIN1* (**C**) across days 0 (grey), 1 (blue), and 2 (green). Vitamin D_3-responsive enhancer and TSS regions are shaded in light grey. Vitamin D target genes are highlighted in red. Tracks show merged data from three biological replicates.
