# Supplementary material for: Chromatin and transcriptional dynamics underlying the immune-modulatory effects of vitamin D3 in vivo
Source: Sci Rep. 2025 Dec 18;16:2997. doi: 10.1038/s41598-025-32831-z (PMC12830676; doi:10.1038/s41598-025-32831-z)
Supplement: Supplementary file 9 — Supplementary Information 9. [file 41598_2025_32831_MOESM9_ESM.pdf]

Short Protocol Template

A. VitDHiD – N-of-1 Repeated Measures Study

Title: VitDHiD – High-Dose Vitamin D_3 Intervention in a High-Responder Individual
Registration: ClinicalTrials.gov NCT03537027
Design: Non-randomized, repeated-measures, N-of-1 interventional study.
Objective: Investigate epigenomic and transcriptomic changes following high-dose vitamin D_3 supplementation in a high-responder individual.
Participant: 59-year-old healthy male; previously phenotyped as high responder.
Intervention: 80,000 IU oral vitamin D_3 bolus, monthly for 3 months.
Sample Collection: Blood at baseline (day 0), 24 h (day 1), 48 h (day 2) post-bolus; repeated for each bolus.
Endpoints: ATAC-seq and RNA-seq changes in PBMCs.
Ethics: Approved by Northern Savo Hospital District Ethics Committee (approval no. 515/2018); written consent obtained.

B. VitDPAS – Non-Randomized Interventional Cohort Study

Title: VitDPAS – Vitamin D_3 Bolus Intervention in a Polish Cohort
Registration: ClinicalTrials.gov NCT06104111
Design: Non-randomized, single-dose, interventional cohort study.
Objective: Investigate population variability in epigenomic and transcriptomic responses to vitamin D_3 bolus.
Participants: 13 healthy adults (5 females, 8 males); responders classified as high (n=4), mid (n=5), and low (n=4).
Intervention: Single body weight-adjusted bolus of 1,000 IU/kg vitamin D_3.
Sample Collection: Blood at baseline (day 0) and 24 h (day 1) post-supplementation.
Endpoints: ATAC-seq (chromatin accessibility) and RNA-seq (gene expression).
Ethics: Approved by Ethics Committee of the Olsztyn Chamber of Physicians (approval no. 31/2023/VIII); written consent obtained.
