# Supplementary material for: Chromatin and transcriptional dynamics underlying the immune-modulatory effects of vitamin D3 in vivo
Source: Sci Rep. 2025 Dec 18;16:2997. doi: 10.1038/s41598-025-32831-z (PMC12830676; doi:10.1038/s41598-025-32831-z)
Supplement: Supplementary file 10 — Supplementary Information 10. [file 41598_2025_32831_MOESM10_ESM.pdf]

STROBE Checklist – VitDPAS (Non-Randomized Interventional Cohort)

Section	Item	Addressed in Manuscript
**Title and Abstract**	Clearly identifies as a non-randomized interventional cohort study.	Abstract, Title
**Introduction**	Background and rationale explained.	Introduction
**Methods**		
Study design	Described as non-randomized interventional.	Methods
Setting	Recruitment in Olsztyn, Poland, 2023.	Methods
Participants	13 healthy adults (5 females, 8 males; high, mid, low responders).	Methods
Variables	Serum 25(OH)D, ATAC-seq profiles.	Methods
Data sources/measurements	Blood collection at baseline (d0) and 24 h (d1) post-supplementation.	Methods
Bias	Acknowledged as non-randomized with potential selection bias.	Discussion
Study size	Fixed by availability of participants.	Methods
Quantitative variables	Serum concentrations, chromatin accessibility, transcriptomic expression.	Results
Statistical methods	DiffBind and DESeq2 pipelines described.	Methods
**Results**		
Participants	Flow chart not required; all 13 completed.	Results
Descriptive data	Baseline characteristics described.	Results
Outcome data	Differential chromatin and gene expression.	Results
Main results	Inter-individual variability discussed.	Results, Discussion
**Discussion**	Limitations, interpretation, and generalizability.	Discussion
**Funding**	Sources listed.	Funding
